# Women’s subjective perceptions and background factors associated with poor maternal childbirth experience among induced and spontaneous onset of labour: a two-year tertiary hospital cohort study

**DOI:** 10.1186/s12884-023-05665-8

**Published:** 2023-05-13

**Authors:** Katariina Place, Leena Rahkonen, Katti Adler, Heidi Kruit

**Affiliations:** Department of Obstetrics and Gynaecology, University of Helsinki and Helsinki University Hospital, HUS, Haartmaninkatu 2, 00029 Helsinki, Finland

**Keywords:** Childbirth experience, Induction of labour, Spontaneous onset or labour, Visual analogue scale, Maternal perception, Maternal satisfaction

## Abstract

**Background:**

Women undergoing induction of labour (IOL) more often have poor childbirth experience compared to women with spontaneous onset of labour (SOL). For understanding and optimizing childbirth experience in IOL, we investigated the subjective maternal reasons and perceptions leading to poor childbirth experience in IOL compared to SOL, as well as the background factors and delivery outcomes associated with the poor experience.

**Methods:**

Two-year retrospective cohort study included 836/19442 deliveries (4.3%) with poor childbirth experience in induced or spontaneous onset at term in Helsinki University Hospital. Poor childbirth experience occurred in 389/5290 (7.4%) cases of IOL and in 447/14152 (3.2%) of SOL. Childbirth experience was measured after delivery using Visual Analog Scale (VAS) score, with poor experience defined as VAS < 5. The primary outcome of the study were the maternal reasons for poor childbirth experience. The parameters were collected in the hospital database and statistical analyses were performed by using Mann–Whitney U-test and t-test.

**Results:**

The subjective maternal reasons for poor childbirth experience were pain (*n* = 529, 63.3%), long labour (*n* = 209, 25.0%), lack of support by care givers (*n* = 108, 12.9%), and unplanned caesarean section (CS) (*n* = 104, 12.4%). The methods of labour analgesia were similar among the women who expressed pain as the main reason compared with those who didn’t. When comparing the reasons according to the onset of labour, IOL group more often reported unplanned CS (17.2% vs. 8.3%; *p* < 0.001) and lack of support by the care givers (15.4% vs. 10.7%; *p* = 0.04), while SOL group more often named pain (68.7% vs. 57.1%; *p* = 0.001) and rapid labour (6.9% vs. 2.8%; *p* = 0.007). In multivariable logistic regression model, IOL was associated with lower risk for pain compared to SOL (adjusted OR 0.6, 95%CI 0.5–0.8; *p* < 0.01). Primiparas more often reported long labour (29.3% vs. 14.3%; *p* < 0.001) and concern over own or baby’s wellbeing (5.7% vs. 2.1%; *p* = 0.03) compared to multiparas. Women who feared childbirth more often reported lack of support compared to women with no fear (22.6% vs. 10.7%; *p* < 0.001).

**Conclusion:**

The main reasons for poor childbirth experience were pain, long labour, unplanned CS and the lack of support by care givers. The childbirth experience is complex and could be optimized by information, support and presence of care givers especially in induced labour.

## Background

Childbirth experience greatly affects the mother´s health and future family planning [1, 2]. Induction of labour (IOL) is a risk factor for a poor childbirth experience, and it is also associated with an experience of greater pain during delivery, higher caesarean section (CS) rate and longer labour duration [3–5]. Also, primiparity, CS, operative vaginal delivery, and maternal complications, such as infection and postpartum haemorrhage, are associated with poor childbirth experience [6–8]. Considering the increasing rates of IOL, being currently approximately one third of all deliveries in developed countries [9–11], understanding the reasons for poor childbirth experience associated with induced labour is of importance.

The aim of this study was to investigate the subjective maternal reasons and perceptions leading to poor childbirth experience in induced labour compared to labour of spontaneous onset, as well as the background factors and delivery outcomes associated with the poor experience.

## Material and methods

This retrospective cohort study was conducted at the department of Obstetrics and Gynaecology, Helsinki University Hospital, between 1.1.2017 and 31.12.2018. We included all women with live singleton pregnancies in cephalic presentation at or beyond 37 gestational weeks who had induced or spontaneous onset of labour, and a poor maternal childbirth experience score. A study population of 836 women with the mean age 31.7 (5.0 SD), the mean body mass index (BMI) 23.9 (3.9 SD) and the mean gestational age of 40.5 (1.2 SD) weeks were included in the study. The rate of primiparous women in the study population was 71.5% (*n* = 598). The study protocol was approved by the institutional review board (IRB) of the hospital region Helsinki and Uusimaa Hospital District Committee for Obstetrics and Gynaecology (nr. HUS/3172/2018 and HUS/54/2019). Due to the retrospective nature of the study, written informed consent was waived by the IRB according to national legislation (Medical Research Act 488/1999, Ch.2a (23.4.2004/295), section 5 and 10a). All methods were carried out in accordance with the Declaration of Helsinki, with the relevant guidelines and regulations.

The primary outcomes of the study were the subjective reasons for the poor childbirth experience as reported by the women themselves. The secondary outcomes were the background factors and labour outcomes associated with poor childbirth experience. Our interest was especially in women with the following risk factors: IOL, primiparity, and fear of childbirth.

The women rated their satisfaction with the subjective childbirth experience in the post-partum ward prior to being discharged using a visual analogue (VAS) score [3]. The women scored their satisfaction with childbirth experience on a scale from zero to ten, with zero representing the most negative experience and ten representing the most positive experience possible. Poor maternal childbirth experience was defined as VAS score < 5 [3]. The women also named reasons for their given score and they could report more than one reason. The maternal reasons for the poor childbirth experience were then categorized as following: pain, long labour, rapid labour, operative vaginal delivery, caesarean delivery, delivery complications, neonatal adverse outcome as perceived by the mother, separation from the baby, fear for own or the baby’s wellbeing with no actual medical reason, expectations not being met, fatigue, unpleasant facilities or environment during labour, lack of support or unsupportive staff, and overall negative experience with no specific factors mentioned. The mother’s experience was also categorized as overall negative experience if the mother felt she wasn`t admitted to the delivery ward on time, or if she gave a VAS score < 5 but declined to further explain the reasons. Separation of the mother and baby included both maternal and neonatal reasons, such as admission to intensive care or need for treatment or monitoring causing the separation.

We collected the data for baseline characteristics and labour outcomes from the hospital electronic database. The collected maternal parameters included gestational age at the time of delivery, parity, maternal age, height, weight, pre-pregnancy body mass index (BMI), smoking history, use of in vitro fertilization (IVF), gestational or pre-gestational diabetes, diagnosis of a psychiatric illness documented, drug or alcohol abuse documented, underprivileged socioeconomic statuss and social worker care documented, fear of labour documented during pregnancy, method for labour induction, oxytocin induction and augmentation, induction to delivery interval, method of pain relief, mode of delivery, indication for CS, shoulder dystocia, post-partum haemorrhage, episiotomy, grade III-IV perineal tear, placental retention, intrapartum and postpartum infection, and severe maternal labour complications including hysterectomy, relaparotomy, urinary bladder injury, and bowel injury. The collected neonatal parameters were malformation of the fetus, gender, birth weight, Apgar score, umbilical artery blood gas values, and admission to neonatal care.

Advanced maternal age was considered as age ≥ 37 years at the time of delivery. Obesity was defined as BMI ≥ 30. Gestational weeks ≥ 41 defined post-term pregnancy. Gestational diabetes was defined as one or more pathological value in a two-hour oral glucose tolerance test. Failed labour induction was defined as at least 12 h of oxytocin administration with ruptured membranes and cervical dilation < 6 cm with no change. Labour arrest was defined as cervical dilation of 6 cm or more, adequate contractions and no change in dilation or descent. Shoulder dystocia was defined as delivery that required special obstetric manoeuvres to deliver the fetus after delivery of the head. Postpartum haemorrhage was defined as blood loss ≥ 1000 ml. Psychiatric illness was defined as a psychiatric diagnosis prior to or during pregnancy with or without pharmacological treatment. Women of an underprivileged socioeconomic status or with a history of alcohol or drug abuse attended regular maternity unit and social worker appointments during pregnancy. Fear of childbirth was defined as either a referral from maternity clinic due to fear or concerns over childbirth, or midwife or doctor’s consultation during pregnancy due to fear of childbirth.

IOL was started by cervical ripening with misoprostol tablets or balloon catheter, and continued by artificially rupturing the membranes and administering oxytocin. Oxytocin augmentation and fetal monitoring with continuous cardiotocography were routinely used during labour.

IBM SPSS Statistics for Windows, Version 26.0 (Armonk, NY, USA) was used for statistical analyses. Chi-square test and Fisher`s exact test were used for comparing categorical variables when appropriate. Continuous variable analyses were performed using Mann–Whitney U test when the assumption of normal distribution was violated, otherwise t-tests were used. A multivariable logistic regression model was performed for calculating adjusted odd ratios (OR) with 95% confidence intervals (CI) for the primary outcome by modelling the data to control for parity, maternal age, maternal pre-pregnancy BMI, onset of labour, fear of childbirth and psychiatric illness. Statistical significance was defined as a *p*-value < 0.05.

## Results

A total of 836 women, 4.3% of all the 19 442 deliveries during the study period were included. In induced labour poor childbirth experience occurred in 389/5290 deliveries (7.4%) and in SOL in 447/14152 deliveries (3.2%). The mean maternal age in was 32.2 years (5.3 SD) in the IOL group and 31.3 years (4.9 SD) in the group of SOL; *p* < 0.001. The rates of primiparity were similar in both induced and spontaneous labour (Table 1). The women in the IOL group were older, more obese, and more often had IVF pregnancy, gestational diabetes, and more advanced gestational age (the mean gestational age 40.7 [1.3 SD] weeks in IOL vs. 40.3 [1.0 SD] weeks in SOL; *p* < 0.001) (Table 1).

**Table 1:** Characteristics of the women with poor childbirth experience and singleton term delivery following induction or spontaneous onset of labour in Helsinki University Hospital over 2017–2018, *N* = 836

	**Induced labour**		**Spontaneous onset of labour**		
	***n* = 389**	**%**	***n* = 447**	**%**	***p*-value**
Primiparous	278	71.5	320	71.6	0.97
Age < 25 years	32	8.2	51	11.4	0.13
Age 25–37 years	281	72.2	337	75.4	0.11
Age ≥ 37 years	76	19.5	59	13.2	**0.01**
BMI ≥ 30	79	20.3	53	11.9	**< 0.01**
In vitro fertilisation	32	8.2	19	4.3	**0.02**
Smoking	36	9.3	43	9.6	0.86
Post-term pregnancy (≥ 41 weeks)	46	11.8	40	8.9	0.17
Gestational diabetes	102	26.2	88	19.7	**0.03**
Previous caesarean section	29	7.5	34	7.6	0.93
Fear of labour	82	21.1	73	16.3	0.08
Psychiatric illness	43	11.1	34	10.3	0.72
Medical treatment for psychiatric illness	25	6.4	21	4.7	0.27
Drug or alcohol abuse during pregnancy	7	1.8	7	1.6	0.79
Underprivileged socioeconomic status	16	4.1	14	3.1	0.45

Psychiatric illness was diagnosed in 77 (9.2%) women prior to or during pregnancy, with no difference between the groups of IOL and SOL (Table 1). The most frequent diagnosis was depression (5.5% [*n* = 22] in IOL vs. 4.3% [*n* = 19] in SOL; *p* = 0.35, respectively). The other psychiatric diagnoses were anxiety (*n* = 18), attention-deficit/hyperactivity disorder (ADHD) (*n* = 7), panic disorder (*n* = 17), post-traumatic stress disorder (*n* = 4), insomnia (*n* = 4), eating disorder (*n* = 4), bipolar disorder (*n* = 4), dissociative disorder (*n* = 8), obsessive compulsive disorder (*n* = 3), psychotic disorder (*n* = 1), combination of two or more of the previous conditions (*n* = 7). Of the 77 women diagnosed with psychiatric illness, 46 women (59.7%) had medical treatment (Table 1). Fear of childbirth was diagnosed in 18.5% (*n* = 155) of the women, underprivileged socioeconomical status was recorded in 3.6% (*n* = 30) of the women, and drug or alcohol abuse during pregnancy were noted in 1.7% (*n* = 14) of the women. None of these factors differed between the groups of women with IOL and SOL (Table 1).

Caesarean delivery and severe labour complication (including hysterectomy, relaparotomy, urinary bladder or bowel injury) were more frequent among women with IOL compared to SOL (Table 2). Thirty-two women (3.8%) underwent emergency CS after a failed attempt of operative vaginal delivery by vacuum extraction, and 11 (34.4%) of these women reported the unsuccessful vacuum attempt as the main cause for poor childbirth experience. Of the 223 women with operative vaginal delivery, 52 (23.3%) reported the procedure as the reason for poor childbirth experience. The mean birthweight was greater in the IOL group (3656 g [488 SD] vs. 3582 g [451 SD]; *p* = 0.02). Neonatal outcomes did not differ between the groups (Table 2).

**Table 2:** Delivery outcomes of the women with poor childbirth experience and singleton term delivery following induction or spontaneous onset of labour in Helsinki University Hospital over 2017–2018, *N* = 836

	**Induced labour**		**Spontaneous onset of labour**		***p*-value**
	***n* = 389**	**%**	***n* = 447**	**%**	
Caesarean section	174	44.7	89	19.9	**< 0.01**
Operative vaginal delivery	83	21.3	140	31.3	0.91
Episiotomy	56	14.4	97	21.7	**< 0.01**
Grade III -IV perineal tear	15	3.9	11	2.5	0.25
Retention of placenta	13	3.3	13	2.9	0.72
Shoulder dystocia	6	1.5	1	0.2	0.05
Post-partum haemorrhage ≥ 1000 ml	94	24.2	86	19.2	0.08
Severe labour complication^a^	7	1.8	1	0.2	**0.03**
Male	202	51.9	242	54.1	0.52
Fetal anomaly	3	0.8	1	0.2	0.34
Macrosomia (≥ 4500 g)	15	3.9	12	2.7	0.34
Apgar 5 min < 7	28	7.2	28	6.3	0.73
Umbilical artery pH < 7.05	11	2.8	8	1.8	0.36
Umbilical artery BE < -12.0	11	2.8	9	2.0	0.51
Neonatal intensive care unit admission	50	12.9	46	10.3	0.25
Induced hypothermia for fetal asphyxia	1	0.3	0	0	

^a^Including hysterectomy, relaparotomy, urinary bladder or bowel injury

**Fig. 1:**
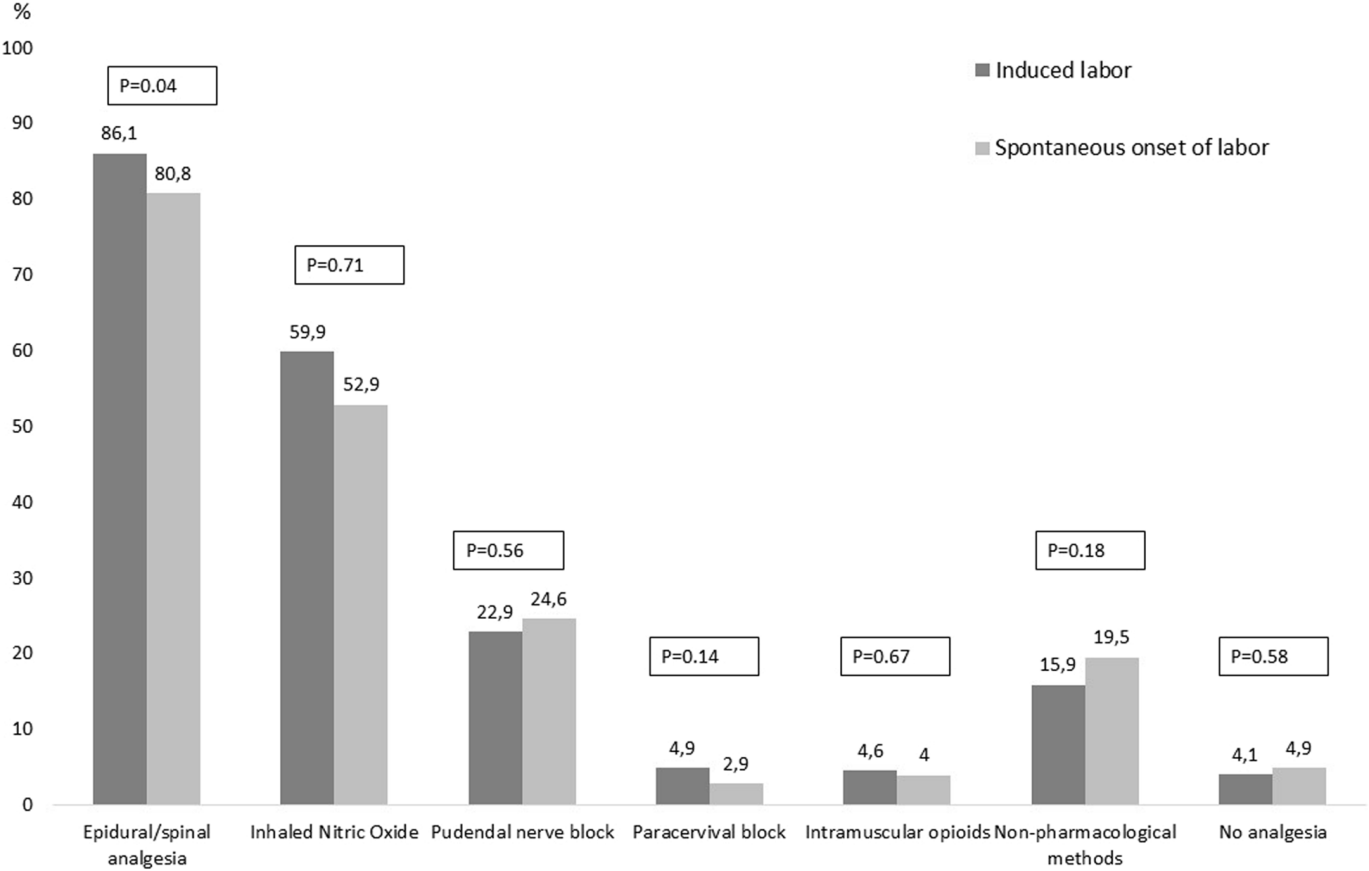
Labour analgesia in induced and spontaneous onset of labour in women with poor childbirth experience (*N* = 836)

The labour analgesia methods are presented in Fig. 1. Thirty-eight women (4.5%) had no pharmaceutical pain relief. Epidural and/or spinal analgesia were more frequent in women with IOL compared with SOL (Fig. 1).

Table 3 presents the subjective reasons for the poor childbirth experience as reported by the women. The overall main reasons were labour pain (*n* = 529, 63.3%), long labour (*n* = 209, 25.0%), lack of support or unsupportive behaviour of care givers (*n* = 108, 12.9%) and unplanned caesarean delivery (*n* = 104, 12.4%) (Table 3).

**Table 3:** The subjective reasons for poor childbirth experience as reported by the women with singleton term delivery following induction or spontaneous onset of labour in Helsinki University Hospital during 2017–2018, *N* = 836

	**n**	**%**
Pain	529	63.3
Long labour	209	25.0
Lack of support or unsupportive staff	108	12.9
Emergency caesarean delivery	104	12.4
Expectations not being met	67	8.0
Delivery complications	63	7.5
Operative vaginal delivery	52	6.2
Neonatal adverse outcome as perceived by the mother	52	6.2
Overall negative experience^a^	50	6.0
Fear for the baby’s or own wellbeing with no medical reason	46	5.5
Rapid labour	42	5.0
Fatigue	18	2.2
Separation from the baby	9	1.1
Unpleasant facilities/environment during labour	7	0.8

^a^The category overall negative experience includes subcategories of “Did not want to specify the reason” *n* = 22 and “Not admitted on delivery ward on time” *n* = 7

Table 4 shows the main reasons for poor childbirth experience in IOL compared with SOL, as well as in primiparous compared with multiparous women, and in women with fear of childbirth compared with no fear reported. Women with SOL more often reported pain and too rapid delivery as the reasons for poor childbirth experience, while women undergoing IOL more often reported unplanned CS, and the lack of support or unsupportive behaviour of the care givers as their reasons for poor childbirth experience (Table 4). Primiparous women more often reported long labour duration while multiparous women more often considered rapid labour as the reason for poor childbirth experience (Table 4). The primiparous women who experienced prolonged labour did have significantly longer median duration of labour compared with those who did not report prolonged labour as the reason (15.7 h [IQR 10.8–22.4 h] vs. 12.9 h [8.8 -17.8 h0]; *p* = 0.001). The median duration of labour for the multiparous women who reported rapid labour as the main cause for poor childbirth experience was significantly shorter compared with the multiparous women who did not mention duration of labour as reason (3.9 h (IQR 2.8 – 6.3 h) vs 7.0 h (IQR 4.3 – 11.3 h); *p* = 0.002). Women who feared childbirth more often felt that the support and supportive behaviour of the care givers was lacking compared with women who had no fear of childbirth (Table 4).

**Table 4:** Reasons for poor childbirth experience as reported by the women in Helsinki University Hospital during 2017–2018, analysed according to type of labor onset, parity, and fear of childbirth, *N* = 836

	**Type of labour onset**					**Parity**					**Fear of childbirth**				
	**Induced**		**Spontaneous**		***p*-value**	**Primiparous**		**Multiparous**		***p*-value**	**Fear**		**No fear**		***p*-value**
**Reason for low VAS-score on childbirth satisfaction**	***n* = 389**	**%**	***n* = 447**	**%**		***n* = 598**	**%**	***n* = 238**	**%**		***n* = 155**	**%**	***n* = 681**	**%**	
Pain	222	57.1	307	68.7	**< 0.01**	382	63.9	147	61.8	0.57	88	56.8	441	64.8	0.06
Long labour	91	23.4	118	26.4	0.32	175	29.3	34	14.3	**< 0.01**	37	23.9	172	25.3	0.72
Rapid labour	11	2.8	31	6.9	**< 0.01**	19	3.2	23	9.7	**< 0.01**	10	6.5	32	4.7	0.37
Operative vaginal delivery	19	4.9	33	7.4	0.14	31	5.2	9	5.2	0.39	8	5.2	32	4.7	0.81
Caesarean delivery	67	17.2	37	8.3	** < 0.01**	81	13.5	23	9.7	0.13	18	11.6	86	12.6	0.73
Delivery complication	30	7.7	33	7.4	0.86	42	7.0	21	8.8	0.37	11	7.1	52	7.6	0.82
Neonatal adverse outcome	26	6.7	27	6.0	0.70	37	6.2	16	6.7	0.77	12	7.7	41	6.0	0.43
Separation from the baby	4	1	5	1.1	0.90	6	1	3	1.3	0.75	2	1.3	7	1	0.78
Fear for the baby’s or own wellbeing with no medical reason	15	3.9	24	5.9	0.30	34	5.7	5	2.1	**0.03**	10	6.5	29	4.3	0.24
Expectations not being met	33	8.5	34	7.6	0.64	46	7.7	21	8.8	0.59	12	7.7	55	8.1	0.89
Fatigue	9	2.3	9	2.0	0.77	16	2.7	2	0.8	0.10	3	1.9	15	2.2	0.84
Unpleasant facilities/environment during labour	3	0.8	4	0.9	1.00	6	1	1	0.4	0.68	2	1.3	5	0.7	0.49
Lack of support or unsupportive staff	60	15.4	48	10.7	**0.04**	76	12.7	32	13.4	0.78	35	22.6	73	10.7	**< 0.01**
Overall negative experience	29	7.5	21	4.7	0.09	34	5.7	16	6.7	0.57	5	3.2	45	6.6	0.11

The methods of labour analgesia were similar among the women who expressed labour pain as the reason for poor childbirth experience compared with the women who didn`t consider pain as the reason (Table 5).

**Table 5:** Type of labour analgesia among the women who reported labour pain as the reason for their poor childbirth compared to women who reported other factors as the reason for poor childbirth experience in Helsinki Universtiy Hospital during 2017–2018, *N* = 836

	**Pain reported as the main reason**		**Reasons other than pain reported as the main reason**		***p*-value**
	***n* = 529**	**%**	***n* = 307**	**%**	
Epidural/spinal analgesia	432	81.7	264	86.0	0.11
Pharmaceutical pain relief	21	4	15	4.9	0.53
Paracervical block	21	4	11	3.6	0.78
Pudendal nerve block	137	25.9	62	20.0	0.06
Non-pharmaceutical pain relief	93	17.6	56	18.2	0.81
No pain relief method	21	4.0	17	5.5	0.29

In the multivariable logistic regression model, women undergoing IOL were at lower risk for experiencing pain as the reason for poor childbirth experience (adjusted OR 0.6, 95%CI 0.5–0.8; *p* < 0.01), but IOL was associated with an increased risk for experiencing emergency caesarean delivery as the reason for poor childbirth experience (Table 6). Primiparity was associated with the risk for experiencing long labour as the reason for poor childbirth experience, and advanced maternal age was associated as the risk for experiencing lack of support from the staff (Table 6).

**Table 6:** Multivariable logistic regression analysis on the risk factors for the main reasons for poor childbirth experience as reported by the women in Helsinki University Hospital during 2017–2018, *N* = 836

		**Pain**			**Long labour**			**Unplanned CS**			**Lack of support by care givers**		
**Factor**	**n**	**OR**	**95% CI**	***p*-value**	**OR**	**95% CI**	***p*-value**	**OR**	**95% CI**	***p*-value**	**OR**	**95% CI**	***p*-value**
Primiparity	598	0.7	0.8–1.5	0.72	**2.6**	**1.7–4.0**	** < 0.01**	1.4	0.9–2.3	0.17	1.1	0.6–1.9	0.87
Induction of labour	**398**	**0.6**	**0.5–0.8**	**< 0.01**	0.8	0.6–1.1	0.23	**2.3**	**1.5–3.6**	**< 0.01**	1.2	0.7–2.1	0.42
Age ≥ 37 years	135	0.8	0.5–1.1	0.21	1.3	0.8–2.1	0.19	0.9	0.5–1.6	0.17	**2.2**	**1.2–4.1**	**0.01**
BMI ≥ 30	132	1,1	0.8–1.7	0.57	1.2	0.8–1.8	0.45	1.0	0.6–1.8	0.94	1.3	0.7–2.6	0.41
Fear of childbirth	155	0.8	0.5–1.1	0.15	1.1	0.7–1.6	0.77	0.8	0.5–1.5	0.52	1.6	0.9–3.0	0.13
Psychiatric illness	77	0.8	0.5–1.3	0.48	0.6	0.4–1.1	0.12	1.4	0.7–2.6	0.32	1.2	0.5–2.6	0.68

## Discussion

In this two-year tertiary hospital cohort study, 4.3% of the women had poor childbirth experience. The main reasons for poor experience, as reported by the women, were pain, long labour, unplanned CS, and lack of support by care givers. Women with IOL more often reported unplanned CS and lack of support by the care givers as their reason, while women with SOL more often named pain and rapid labour. Primiparas more often reported long labour and concern over own or baby’s wellbeing as the reason compared to multiparas. The women who named long or rapid labour as the main reason for their poor childbirth experience also had statistically significantly longer or faster labours compared with the average labour durations in the study population, while labour pain was a more subjective experience. The labour analgesia methods and frequency were similar between the women who named pain as the main reason and those who didn’t. IOL was associated with lower risk for experiencing pain as the reason for poor childbirth experience compared to SOL. Women with fear of labour more often reported lack of support or unsupportive behaviour by the treating staff compared to women without fear.

Previous studies indicate that women whose labour is induced experience more pain than women with spontaneous onset of labour [11]. On the contrary, in our study the women with SOL more often reported pain as the reason for poor childbirth experience compared with women who underwent IOL. Furthermore, higher labour pain scores have previously been documented in nulliparous women, especially if there has been no antenatal education [12]. In our study, however, there was no difference in the frequency of labour pain reported as the reason for poor childbirth experience between the primiparous and parous women. Among all women who expressed pain as the reason for poor childbirth experience, no difference in the pain relief methods or frequency was seen compared with those who didn’t report pain as a reason for poor childbirth experience. However, we often focus on offering efficient pain relief in hope of improving the labour experience. Some previous studies have reported pain relief playing only a minor role in women’s satisfaction with the childbirth [7, 13], which is supported by our study. It has also been suggested that the most effective strategies to create a positive childbirth experience are supporting women during birth, providing intrapartum care with minimal intervention, birth preparedness and improving readiness for complications [8, 14–16]. These findings are supported by also the current study.

Women with induced labour and women with fear of childbirth prior to labour more often reported the lack of support or unsupportive behaviour of the staff as the reason for poor childbirth experience compared with spontaneous onset of labour and women with no reported fear of childbirth. This emphasises the fact that childbirth is a complex emotional experience involving both physiological and psychological mechanisms. We find that since perception of pain in the study population was subjective with no correlation to the method or frequency of the analgesia received, more focus should be put on intrapartum support and presence by care givers.

We found that the women with IOL were less satisfied when their delivery was by emergency CS, compared with women with SOL and emergency CS. Similar results have previously been reported in the studies by Waldenström [7] and Dupont [17]. Interestingly, adverse neonatal outcome and separation from the newborn did not play a major role in the reasons for poor childbirth experience as reported by the parturient.

The retrospective nature of the study may be seen as a strength since it reflects the reality of the labour ward, whereas in a prospective study the care givers could be more self-conscious of their behaviour and find more time for the women during the study. The VAS score being collected at the post-partum ward prior to discharge by different midwife in an unstandardized way of communicating can be seen as a limitation of the study. Without leading questions or sufficient time for discussion, the mother might have left some things unsaid although these may have played a significant role in developing the poor childbirth experience. Another weakness of the study is that the VAS score was asked soon after delivery. According to earlier studies, the perception of childbirth and labour pain may change significantly over time [18]. Hence, it would be useful to investigate the childbirth experience after a longer period from delivery. It could also be helpful in finding the group of women who would benefit from additional support.

In conclusion, the childbirth experience is complex and subjective. Several factors, such as pain, long labour, unplanned CS and lack of support by care givers, affect the woman's perception of childbirth making each experience unique. Sufficient information, support and presence by care givers are important in improving the childbirth experience especially in induced labour.

## Data Availability

The datasets analysed under license for the current study are not publicly available due to the restrictions applied to the availability of these data by the Helsinki and Uusimaa Hospital District. Data are however available from the corresponding author on reasonable request.

## Abbreviations

BMI: Body mass index
CS: Caesarean section
IOL: Induction of labour
SOL: Spontaneous onset of labor
